# The genetics of circadian rhythms, sleep and health

**DOI:** 10.1093/hmg/ddx240

**Published:** 2017-07-14

**Authors:** Aarti Jagannath, Lewis Taylor, Zeinab Wakaf, Sridhar R Vasudevan, Russell G Foster

**Affiliations:** 1 Sleep and Circadian Neuroscience Institute, OMPI-G, Sir William Dunn School of Pathology, University of Oxford, Oxford OX1 3RE, UK; 2 Department of Pharmacology, University of Oxford, Mansfield Road, Oxford OX1 3QT, UK

## Abstract

Circadian rhythms are 24-h rhythms in physiology and behaviour generated by molecular clocks, which serve to coordinate internal time with the external world. The circadian system is a master regulator of nearly all physiology and its disruption has major consequences on health. Sleep and circadian rhythm disruption (SCRD) is a ubiquitous feature in today’s 24/7 society, and studies on shift-workers have shown that SCRD can lead not only to cognitive impairment, but also metabolic syndrome and psychiatric illness including depression (1,2). Mouse models of clock mutants recapitulate these deficits, implicating mechanistic and causal links between SCRD and disease pathophysiology (3–5). Importantly, treating clock disruption reverses and attenuates these adverse health states in animal models (6,7), thus establishing the circadian system as a novel therapeutic target. Significantly, circadian and clock-controlled gene mutations have recently been identified by Genome-Wide Association Studies (GWAS) in the aetiology of sleep, mental health and metabolic disorders. This review will focus upon the genetics of circadian rhythms in sleep and health.

## Introduction to the Circadian Clock

**Figure 1.**
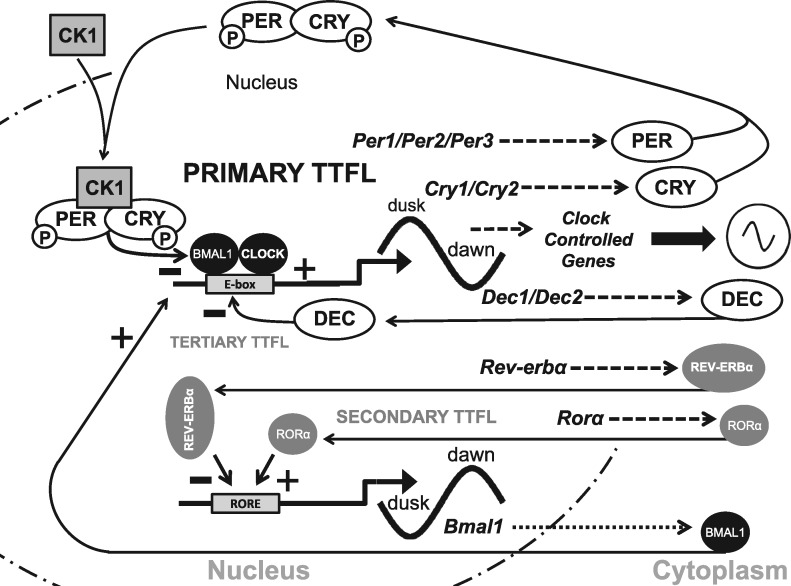
The mammalian molecular clock. The driving force of the mammalian molecular clockwork is the transcriptional drive provided by two proteins named ‘Circadian Locomotor Output Cycles Kaput’, CLOCK (CLOCK), which heterodimerises with ‘Brain muscle arnt-like 1’ (BMAL1). *Bmal1* gene transcription produces a rhythmically produced BMAL1 protein that heterodimerises with a constitutively expressed CLOCK. The CLOCK-BMAL1 complex binds to E-box promoters driving rhythmic transcription of the *Per1-3* and two Cryptochrome genes (*Cry1, Cry2*). The various PER and CRY proteins can complex (dimerise) with themselves to form PER-PER homo- or PER-CRY heterodimers. PER is phosphorylated by the kinase CK1 (Casein kinase 1 family of kinases) and other kinases earmarking it for degradation. However, the PER-CK1 complex allows the CRYs to bind to form a CRY-PER-CK1 complex which prevents further phosphorylation and degradation of PER in the cytoplasm. Within the complex of CRY-PER-CK1, CRY and PER are phosphorylated by other kinases which then allows the CRY-PER-CK1 complex to move into the nucleus and inhibit CLOCK-BMAL1 transcription of the *Per* and *Cry* genes forming the core negative limb of the transcriptional/translational feedback loop (TTFL). The CRY-PER-CK1 protein complex levels rise throughout the day, peak at dusk and decline to their lowest level the following dawn. The stability/degradation rate of the CRY-PER-CK1 complex in the nucleus and the resumption of CLOCK-BMAL1 mediated transcription is a key process in setting the period of the clock. It seems that CK1 and other kinases phosphorylate PER and target it for degradation, whilst at least two F-Box protein (FBXL3 and 11) target CRY proteins for degradation. The net result is that CRY and PER proteins fall to their lowest levels just before dawn. Light acts to up-regulate *Per1* and *Per2* transcription and this allows the entrainment of the molecular clockwork to the dawn/dusk cycle. An interlocked secondary TTFL directs alternating activation and repression of BMAL1 expression. This occurs via the nuclear receptors RORα (RAR-related orphan receptor alpha) and REV-ERBα, respectively, via binding at ROR elements (retinoic acid-related orphan receptor response elements/ROREs) in the *Bmal1* promoter. Both *Rorα* and *Rev*-erbα have an E-box and are driven rhythmically via CLOCK-BMAL1 transcription. The rates of transcription and translation of these genes differ so that ROR peaks at dawn and REV-ERBα peaks at dusk and this action on the Bmal1 promoter ensures that BMAL1 levels rise at dusk, peak at dawn and then fall throughout the day to their low point just before dusk. In this way BMAL1 levels cycle in antiphase to those of CRY and PER. The Dec1 and Dec2 genes give rise to DEC1 and DEC2 proteins which inhibit CLOCK-BMAL1 transcription and constitute the tertiary TTFL, which reinforces the action of CRY-PER-CK1 inhibition on CLOCK-BMAL1 transcription. Finally, the presence of an E-box in the promoter of downstream clock target genes gives rise to overt circadian rhythms in physiology and behaviour. However, it is also known that many clock controlled genes do not possess an E-Box. As a result the nature of the circadian regulation in these genes remains uncertain.

Life has evolved under a 24-h rhythm where environmental factors such as temperature and light fluctuate with a daily predictable sequence. As a consequence, most organisms have evolved circadian clocks that anticipate these regular environmental changes and establish endogenous 24-h rhythms to get the correct physiology and behaviour to the appropriate time window each day. The mechanisms underlying circadian regulation are cell autonomous transcription-translation feedback loops (TTFLs): In mammals, the transcription factors CLOCK and BMAL1 drive the expression of *Period (Per1/2)* and *Cryptochrome (Cry1/2)*, whose protein products in turn feed-back to inhibit CLOCK and BMAL1 (8) (Fig. 1). Downstream of these four factors lie thousands of clock-controlled genes that orchestrate the oscillation of tissue-specific metabolic and physiological functions. Most cells in the body possess a molecular clock and are maintained in synchrony by a master pacemaker located in the suprachiasmatic nuclei (SCN) of the hypothalamus (9).

**Figure 2.**
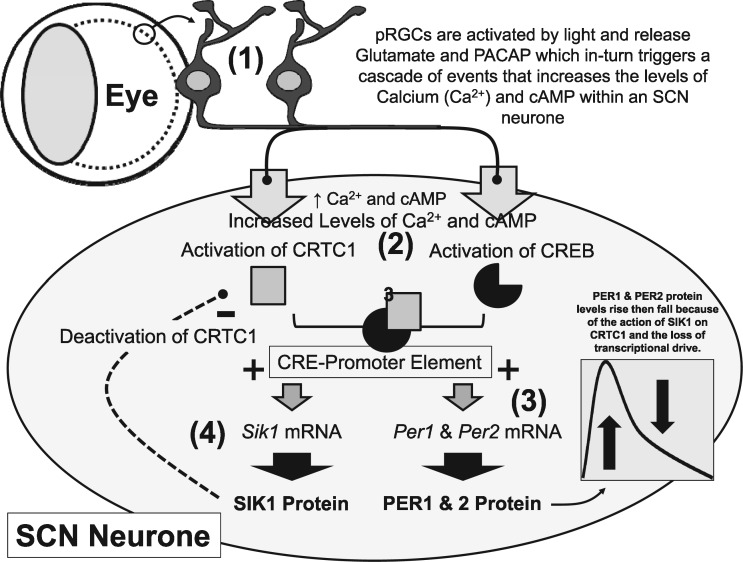
Light regulation of the molecular clockwork in mammals. The sequence of events that entrains the molecular clockwork of a SCN neurone to the solar day are summarised here and involve the following steps: (1) Light is detected by the photosensitive retinal ganglion cells (pRGCs) within the eye. This induces the release of neurotransmitters (glutamate and pituitary adenylate cyclase-activating polypeptide/PACAP) from the pRGC terminals which synapse with neurones in the ventral SCN. These neurotransmitters trigger a sequence of events that increase the levels of Calcium (Ca^2+^) and 3',5'-cyclic adenosine monophosphate (cAMP) within an SCN neurone. Calcium levels rise as a result of influx from the extracellular medium or release from internal stores. (2) Raised intracellular Ca^2+^ and cAMP activate two proteins: CREB-binding protein (CREB) through phosphorylation by Protein Kinase A (PKA) and CREB-regulated transcription coactivator 1 (CRTC1) by dephosphorylation, these work together and bind to a cAMP response element (CRE element) in the promoter of *Per1*, *Per2* and *Sik1*. (3) CRE activation of the *Per* genes (+), leads to elevated *Per* mRNA and increased levels of PER1 and PER2 protein. Changed levels of PER 1 and 2 act to shift the molecular clockwork, advancing the clock at dawn and delaying the clock at dusk. However, *Per* mRNA and PER protein levels fall rapidly even if the animal remains exposed to light. As a result, the effects of light on the molecular clock are limited and entrainment is a gradual process requiring repeated shifting stimuli over multiple days. This phenomenon explains why we get jet-lag, the clock cannot move immediately to a new dawn/dusk cycle because there is a ‘brake’ on the effects of light on the clock. (4) The mechanism that provides this molecular brake is the production of SIK1 protein. SIK1 deactivates CRTC (-) by phosphorylation, so that it can no longer provide the co-transcriptional drive with CREB on the CRE promoter, and transcription largely stops. This negative feedback turns off *Per1* and *Per2* transcription and translation, limiting the effects of light on the clock. *Sik1* mRNA and SIK1 protein levels also decline but more slowly than PER 1 and 2. The system then re-sets itself for possible light detection several hours later. Experiments on mice in which SIK1 has been suppressed show very rapid entrainment to simulated jet-lag. By limiting the shifting effects of light on the SCN, the circadian system of the animal is protected from abnormal light exposure at the wrong time of day. In addition, it may be important to buffer the effects of light on the SCN clock so that it is not pulled rapidly to a new phase, and in the process uncouple the SCN from the peripheral circadian network, resulting in internal desynchrony.

In order for the circadian network to have adaptive value, it must receive and respond to signals that provide temporal cues (zeitgebers). Zeitgebers modulate the temporal expression patterns of clock genes such as *Per1/2* (10), to set the phase, amplitude and period of the molecular clockwork. Light, which signals the dawn-dusk cycle, is the best-characterised zeitgeber, and this light input from the photosensitive retinal ganglion cells (pRGCs) of the retina (11) is transmitted directly to the ventral SCN through synaptic connections, where glutamate signalling then drives cAMP response element binding factor (CREB-CRTC)-mediated transcription of *Per* genes in the SCN (12) (Fig. 2). Peripheral circadian clocks throughout the body receive inputs from the SCN and numerous additional signals, including feeding (13); glucocorticoids (14); temperature (15); and indicators of physiological condition such as metabolic state (16) and sleep history (17,18). The mechanisms by which many of these these zeitgebers interact with the molecular clockwork of the peripheral clocks remains unclear.

Circadian clock outputs have a profound impact upon the biology of a cell, with anywhere between 2 and 30% of each tissue’s transcriptome displaying a circadian rhythm (19–21). Interaction between clock transcription factors and tissue specific transcription factors overlay a circadian rhythm onto tissue specific gene expression patterns (22), resulting in the appropriate circadian transcriptome and in turn, appropriately timed physiology and behaviour. As a result, sleep and circadian rhythm disruption resulting from either social/health reasons or mutations in circadian and clock-controlled genes contributes to the development of a range of disorders. The evidence for these genetic links is discussed below with three examples: mental illness, metabolic disorders and sleep timing disruption. There is also compelling evidence for many other links between circadian disruption and conditions such as cancer (23) and immune system disorders (24), which are not discussed here.

## Circadian Rhythm Disruption in Mental Illness

There is considerable evidence that patients with neuropsychiatric diseases, such as bipolar disorder, schizophrenia and depression exhibit SCRD and this, alongside the evidence from mouse models has been extensively reviewed previously (2,25). This disruption encompasses a wide range of sleep perturbations, including fragmented sleep, reduced total sleep time and changes in normal sleep architecture (26). Furthermore, these patients show dysregulation of multiple circadian outputs and of the core molecular clock (Fig. 1). Remarkably, fibroblasts isolated from schizophrenic patients show a loss of rhythmicity in *CRY1* and *PER1* expression, and their peripheral blood leukocytes have decreased and/or disrupted diurnal expression of *CLOCK*, *PER1*/2/3, *CRY1* and a functional *CLOCK* homologue *NPAS2* in comparison to healthy controls (27). Fibroblasts isolated from bipolar patients display a larger variance in period and amplitude and deficits in the entrainment pathways. Lithium is used for the treatment of bipolar disorder, and lithium’s primary therapeutic target is postulated to be Rev-erba (28) (Fig. 1). Additionally, patients with major depressive disorder display a marked disruption in the circadian rhythmicity and phasing of core clock genes across multiple brain regions (29).

It is becoming increasingly clear that disruption of the molecular clock is not just a consequence of neuropsychiatric illness, but instead forms part of a bidirectional feedback loop with neuropsychiatric disease, whereby perturbations in one exacerbate dysfunction in the other (2,5). In this context, it is worth noting that, many disease relevant processes are under circadian control, such as sleep-wake timing and monoaminergic neurotransmitter synthesis, signalling and degradation (30–32). Furthermore, multiple single nucleotide polymorphisms (SNPs) in the genes encoding the core components of the molecular clock have been demonstrated, albeit weakly, to be associated with schizophrenia, bipolar disorder and depression, suggesting a causal role for clock dysfunction in neuropsychiatric disease (Table 1).

**Table 1:** A list of single nucleotide polymorphisms (SNPs) in core clock genes that are associated with neuropsychiatric or neurodegenerative diseases. Only *P* values highlighted in bold remain significant after multiple comparisons correction

Gene	Disease	Sample size	Total SNPs tested	SNP	*P* value	Test used	Reference
*ARNTL*	BPD	180 controls	44	*rs1481892*	*P = * 0.018	Cochran-Armitage trend test	(33)
		234 patients		*rs4757142*	*P = * 0.0009		
				*rs1982350*	*P = * 0.005		
				*rs7107287*	*P = * 0.033		
	BPD	477 controls	268	*rs7126303*	*P = * 0.04	Cochran-Armitage trend test	(34)
		523 patients					
	SAD	136 controls	13	*rs2290035*	*P = * 0.02	Logistic regression analysis	(35)
		137 patients					
	MD	926 controls	115	*rs2290036*	*P = * 0.043	Logistic regression analysis	(36)
		459 patients					
	PS	913 controls	6	*rs2290036*	*P = * 0.005	Logistic regression analysis	(37)
		535 patients					
	BPD	405 controls	92	*rs3789327*	*P = * 0.0212	Association testing using FBAT	(38)
		465 patients					
	AD	423 controls	1	*rs2278749*	*P < * 0.0001	Pearson’s chi-squared test	(39)
		296 patients					
	PD	1342 controls	125	*rs7950226*	*P = * 0.0088	Cochran-Armitage trend test	(40)
		1394 patients		*rs11605776*	*P = * 0.0049		
				*rs10832022*	*P = * 0.0048		
				*rs11022765*	*P = * 0.0049		
				*rs7941761*	*P = * 0.0197		
				*rs1562437*	*P = * 0.0013		
				*rs3816358*	*P = * 0.0275		
				*rs900147*	*P = * 0.00423*		
*CLOCK*	BPD	101 patients	1	*rs180260*	*P = * 0.026	One-way ANOVA	(41)
	BPD	635 controls	44	*rs180260*	*P = * 0.0138	Association determined using the SNPassoc software package	(42)
		515 patients		*rs11932595*	*P = * 0.0319		
	SZ	128 controls	1	*rs180260*	*P = * 0.026	Logistic regression analysis	(43)
		145 patients					
	SZ	199 controls	1	*rs180260*	*P < * 0.05	Pearson’s chi-squared test	(44)
		145 patients					
	MD	776 controls	32	*rs180260*	*P = * 0.028 (Male patients)	Pearson’s chi-squared test	(45)
		592 patients					
	AD	423 controls	1	*rs180260*	*P < * 0.0001	Pearson’s chi-squared test	(46)
		296 patients					
	BPD	405 controls	92	*rs17777929*	*P = * 0.0317	Association testing using FBAT	(38)
		465 patients					
	BPD	614 controls	62	*rs534654*	*P = * 0.0097	Pearson’s chi-squared test	(47)
		518 patients		*rs4340844*	*P = * 0.015		
				*rs6850524*	*P = * 0.012		
	BPD	444 BPD families	197	*rs6850524*	*P = * 0.032	Pearson’s chi-squared test	(48)
		130 unrelated BPD families		*rs3805148*	*P = * 0.009		
				*rs3736544*	*P = * 0.024		
				*rs12504300*	*P = * 0.009		
				*rs4864542*	*P = * 0.01		
				*rs12648271*	*P = * 0.037		
	BPD	440 controls	209	*rs10462028*	*P = * 0.02	Logistic regression analysis	(49)
		199 patients					
	AD	188 controls	1	*rs1554483*	*P = * 0.009	Pearson’s chi-squared test	(50)
		130 patients					
	AD	423 controls	1	*rs4580704*	*P < * 0.0001	Pearson’s chi-squared test	(51)
		296 patients					
*CRY1*	MD	654 BPD patients	7	*rs10861688*	*P = * 0.0048*	Covariated linear regression	(52)
	MD	440 controls	209	*rs2287161*	***P = * 0.007†**	Logistic regression analysis	(49)
		335 patients					
	MD	485 controls	3	*rs2287161*	*P = * 0.010	Logistic regression analysis	(53)
		105 patients					
*CRY2*	BPD	477 controls	268	*rs1554338*	*P = * 0.031	Cochran-Armitage trend test	(34)
		523 patients					
	MD	1011 controls	4	*rs10838524*	*P = * 0.0017	Logistic regression analysis	(54)
		118 patients		*rs10838327*	*P = * 0.00074		
				*rs3824872*	*P = * 0.007		
	DT	3871 controls	48	*rs10838524*	**q = 0.04**	Linear and logistic regression analysis	(55)
		136 patients		*rs7121611*	**q = 0.04**		
				*rs7945565*	**q = 0.04**		
				*rs1401419*	**q = 0.04**		
	DT	4154 controls	48	*rs10838524*	**q = 0.003**	Logistic regression analysis	(56)
		166 patients		*rs7121611*	**q = 0.002**		
				*rs7945565*	**q = 0.002**		
				*rs1401419*	**q = 0.002**		
				*rs3824872*	**q = 0.02**		
	MD	4154 controls	48	*rs7123390*	**q = 0.05**	Logistic regression analysis	(56)
		862 patients		*rs2292910*	**q = 0.05**		
				*rs7121611*	**q = 0.02**		
				*rs7945565*	**q = 0.02**		
				*rs1401419*	**q = 0.03**		
*NR1D1*	BPD	444 BPD families	197	*rs2071427*	*P* = 0.0019	Pearson’s chi-squared test	(48)
		130 control families		*rs2269457*	*P = * 0.0292		
				*rs2314339*	***P = * 0.0005**		
	PD	1342 controls	125	*rs3744805*	*P = * 0.00294	Cochran-Armitage trend test	(40)
		1394 patients					
*PER1*	PD	1342 controls	125	*rs2253820*	*P = * 0.00067*	Cochran-Armitage trend test	(40)
		1394 patients					
*PER2*	SAD	173 controls	13	*rs10870*	*P = * 0.03	Logistic regression analysis	(35)
		177 patients					
	MD	459 controls	115	*rs2304672*	*P = * 0.0087	Logistic regression analysis	(57)
		926 patients		*rs10462023*	*P = * 0.0033		
				*rs6431590*	*P = * 0.036		
				*rs3739064*	*P = * 0.018		
	SZ	477 controls	268	*rs2304672*	*P = * 0.048	Cochran-Armitage trend test	(34)
		527 patients		*rs2304674*	*P = * 0.033		
	BPD	180 controls	44	*rs2859387*	*P = * 0.039	Cochran-Armitage trend test	(33)
		138 patients					
*PER3*	SZ	180 controls	44	*rs228729*	*P = * 0.028	Cochran-Armitage trend test	(33)
		331 patients					
	SZ	477 controls	268	*rs10462021*	*P = * 0.036	Cochran-Armitage trend test	(34)
		527 patients		*rs2640909*	*P = * 0.031		
	MD	2915 controls	529	*rs12137927*	*P = * 0.00054	Logistic regression analysis	(58)
		1296 patients		*rs228644*	*P = * 0.00013		
				*rs228682*	*P = * 0.00014		
	MD	776 controls	32	*rs17031614*	*P = * 0.017	Pearson’s chi-squared test	(45)
		592 patients		*rs228697*	***P = * 0.007**		
*RORA*	MD	459 controls	115	*rs2028122*	*P = * 0.044	Logistic regression analysis	(57)
		926 patients					
	MD	4811 participants	Whole genome	*rs12912233*	*P = * 6.3 × 10^−7^	Weighted z score-based fixed effects meta-analysis	(59)
				*rs4775340*	*P = * 6.3 × 10^−6^		
				*rs8028646*	*P = * 7.2 × 10^−6^		
				*rs8023563*	*P = *1.5 × 10^−5^		
	MD	2915 controls	529	*rs11632098*	*P = * 0.00056	Logistic regression analysis	(58)
		1296 patients					
	BPD	1759 controls	353	*rs782931*	*P = * 0.01*	Pearson’s chi-squared test	(60)
		479 patients					
	BPD	200 controls	27	*rs4774388*	*P = * 0.024	Additive, dominant and recessive genetic models with a maximum test for associations	(61)
		280 patients					
	BPD	1770 controls	429	43 SNPs reached nominal significance	*P = * 0.002–0.044		
		448 patients					
*RORB*	SZ	477 controls	268	*rs10491929*	*P = * 0.023	Cochran-Armitage trend test	(34)
		527 patients					
	BPD	477 controls	268	*rs17691363*	*P = * 0.035	Cochran-Armitage trend test	(34)
		527 patients		*rs10217594*	*P = * 0.026		
				*rs10491929*	*P = * 0.023		
	PD	1342 controls	125	*rs10491929*	*P = * 0.0264	Cochran-Armitage trend test	(62)
		1394 patients		*rs10869412*	*P = * 0.0097		
				*rs17611535*	*P = * 0.0037		
				*rs17612113*	*P = * 0.0163		
				*rs10521463*	*P = * 0.0068		
	BPD	200 controls	27	*rs1327836*	*P = * 0.003	Additive, dominant and recessive genetic models with a maximum test for associations	(61)
		280 patients		*rs17611535*			
	BPD	1770 controls	429	*rs1761135*	*P = * 0.027		
		448 patients		*rs499922*	*P = * 0.042		

* denotes a Bonferroni corrected *P* value, ^†^ denotes a permutation corrected *P* value. All other *P* values are not adjusted for multiple comparisons. q denotes the false discovery rate q-values, used to correct for multiple comparisons. q < 0.05 was taken to be statistically significant.

*Abbreviations:* AD:  Alzheimer’s disease; BPD: Bipolar disorder; DT:  dysthymia; MD:  major depression; PD:  Parkinson’s disease; PS:  psychosis; SAD:  seasonal affective disorder; SZ:  schizophrenia.

Currently the functional consequence of these SNPs and the strength of their association with disease remains unclear, however, recent work has provided insight into how mutations may impact clock function. Two rare missense mutations in the *PERIOD3* gene (*PER3*-P415A/H417RI), found to be associated with seasonal depression, were demonstrated to generate a mutant PER3 protein unable to stabilise PER1/2 and induce their nuclear localisation, resulting in circadian rhythm disruption (63).

A similar relationship has been found in patients with neurodegenerative diseases. Many conditions are associated with the disruption of sleep, circadian outputs and the core molecular clock (64). Patients with Alzheimer’s disease (AD) exhibit neuronal loss in the SCN (65), and a recent study by Lim *et al.* found that the diurnal and seasonal transcriptional rhythmicity of core clock genes in the dorsolateral prefrontal cortex is disrupted in AD patients (66). In addition, the expression of *BMAL1*/2 is dampened in peripheral blood leukocytes isolated from Parkinson’s disease (PD) patients (67,68).

As with neuropsychiatric illness, disruption of the core molecular clock is both a consequence of, and a contributor towards, neurodegenerative diseases. For example β-amyloid (Aβ), the neuronal aggregation of which is the hallmark of AD, causes BMAL1 degradation and therefore molecular clock disruption (69) (Fig. 1). In animal models it has been shown that sleep deprivation leads to increased Aβ plaque formation and that sleep is required for the clearance of Aβ (70). Additionally, the circadian clock regulates many molecular processes commonly involved in neurodegeneration, such as oxidative stress (71), metabolism (see next section), neuroinflammation (72,73) and protein dynamics (74). Evidence linking SNPs in core clock genes with neurodegenerative diseases is currently scarce, with only a limited number of studies demonstrating the association of SNPs in *CLOCK*, *BMAL1* and/or *PER1* with AD or PD. Collectively, there is currently compelling evidence that disruption of the molecular clock contributes to the progression of both neurodegenerative and neuropsychiatric conditions.

## Metabolic Disorders

**Figure 3.**
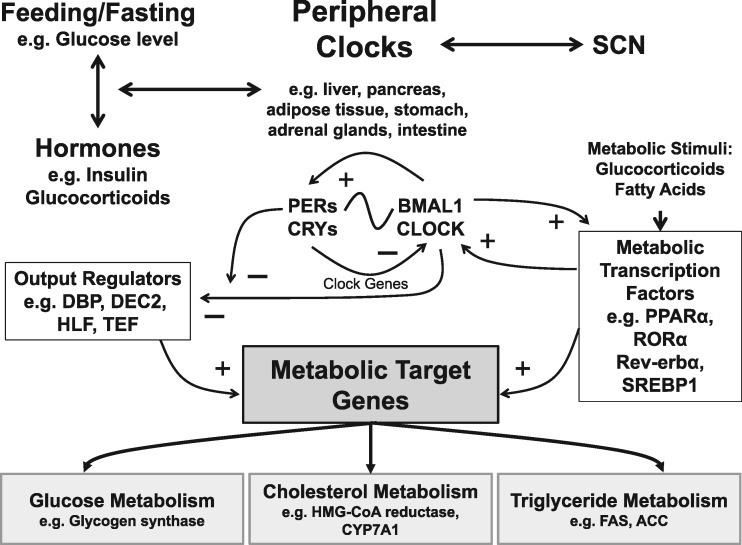
The circadian control of metabolic pathways. Metabolism is under strong circadian control. Peripheral clocks (e.g. liver, pancreas, adipose tissue, etc.) are regulated by the SCN and in turn feedback upon the SCN. Light regulates the phase of the molecular clockwork in the SCN, whilst hormonal signals (e.g. insulin and glucocorticoids) and feeding/fasting behaviours that change the levels of glucose alter the phase of peripheral clocks. The molecular clockwork of both peripheral and SCN cells then interacts with the metabolic control systems. The molecular clock comprises a *Per/Cry* and *Clock/Bmal1* feedback loop (See Figure 1). These genes and their protein products also control the expression of downstream transcription factors which in turn regulate metabolic target genes. General regulators include DBP (D site of albumin promoter (albumin D-box) binding protein), which binds to an upstream promoter in the insulin gene; HLF (Hepatic leukaemia factor), which regulates aspects of liver function; and TEF (Thyrotroph embryonic factor), involved in thyroid-stimulating hormone release. The circadian coordination of metabolism also involves members of the rev-erb (REV-ERB) receptor family, retinoic acid orphan receptors (ROR), PPARs (peroxisome proliferator-activated receptors) and other nuclear receptors (NR). Metabolic regulators, such as REV-ERBα and ROR, also participate directly in the clock mechanism by regulating *Bmal1* transcription (See Figure 1). In addition, hepatic PPARα, which is activated by fatty acids, is regulated rhythmically by CLOCK and BMAL1 and is also regulated by glucocorticoids. These transcriptional regulators in-turn interact with genes associated with glycogen, fatty acid and triglyceride metabolism. Such target genes include: Glycogen synthase, involved in converting glucose to glycogen; HMG-CoA reductase which is the rate-controlling enzyme that produces cholesterol; CYP7A1 is a rate-limiting enzyme in bile acid synthesis; Acetyl-CoA carboxylase (ACC) and Fatty acid synthase (FAS) are involved in catalysing the synthesis of fatty acids. This regulation can be immensely complex, with multiple interlocking feedback loops between the clock and metabolic genes/proteins. For example, transcriptional regulation of rhythmic CYP7A1, is driven by DBP, the clock protein DEC2, and by nuclear receptors including PPARα. PPARα also regulates Rev-erbα expression in both liver and adipose cells, whilst ROR and Rev-erbα regulate lipid metabolism as well as being involved in *Clock* and *Bmal1* expression.

The metabolic system is under strong circadian control, and these relationships are summarised in Figure 3. One of the first indications of the strong coupling between circadian clocks and metabolism was suggested by the observation that the majority of cycling transcripts in the liver are implicated in multiple metabolic pathways (19,75). Processes such as glucose, cholesterol and triglyceride metabolism are a few examples, whose rate-limiting steps were shown to be major sites of circadian regulation.

Clock genes are linked directly to metabolic syndrome (MetS), both in mutant mice and humans. For example, homozygous *Clock* mutant mice (*Clock*^Δ19/Δ19^), which show a loss of function of this core clock gene, are obese and hyperphagic and develop a myriad of metabolic symptoms including hyperglycemia, hyperinsulinemia, hepatic steatosis and dyslipidemia (76), all of which are significant markers of MetS. In addition, impairing *Clock* function in mice suppresses gluconeogenesis and the complete knock out of *Bmal1* gene abolishes it (77). It has also been shown that diabetes mellitus can be triggered by conditional ablation of the *Clock* gene in pancreatic β-cells. *Clock* disruption in pancreatic islets results in transcriptome-wide variations in the expression of genes involved in survival, growth and synaptic vesicle assembly within these cells. Furthermore, *Clock*^Δ19/Δ19^ mutant mice exhibit significant hypoinsulinemia and hyperglycemia as a result of abolishing their pancreatic clocks (4). In addition, *Bmal1* levels have been shown to increase significantly during adipose differentiation in 3T3-L1 mouse embryonic cells and both knock-in and knock-down of *Bmal1* support its critical involvement in adipose differentiation and lipogenesis (78). *Cry1*^-/-^ and *Cry2*^-/-^ mice show no difference in food consumption and body weight compared to wildtype animals, however, when restricted to a high-fat diet, ablation of *Cry1* (yet interestingly not *Cry2*) prevented obesity in these mutant mice (79). Finally, pharmacological induction of RORa transcription factor function, an enhancer of *Bmal1* expression (Fig. 1), has been shown to increase significantly the amplitude of clock rhythms and, remarkably, prevent weight gain in mice fed high-fat diets and attenuate symptoms of MetS (80).

In humans, like mice, polymorphisms of *CLOCK* and *BMAL1* have been associated with metabolic disorders. For example, *Clock* gene polymorphisms have been linked to a higher susceptibility to obesity (81,82) and two haplotypes of *BMAL1* have been associated with hypertension and type 2 diabetes mellitus, replicated both in humans and in rodent models (83). Similar studies have also linked polymorphisms in other core clock genes like *PER2* and *NPAS2* to fasting hyperglycemia and hypertension respectively (84). In a small population of lean and obese women, a correlation between obesity and core clock components has been reported. Remarkably, being obese alters expression of core clock genes in adipocytes throughout the day and induces notable upregulation of *CRY2* and *REV*-*ERBa*, two important negative feedback components of circadian clocks (85) (Fig. 1). Furthermore, a rare SNP in *visfatin* (*NAMPT*/*PBEF1*), a gene known to be involved in the negative arm of the clock (86) (not shown in Figures), has been associated with protection from obesity in human populations (87).

It is now evident that circadian clocks do not only regulate metabolism, but metabolic pathways can in turn feedback upon the circadian clockwork (Fig. 3). Restricting feeding to daytime (sleep phase) in mice causes uncoupling of peripheral clocks within the liver, kidney, heart and pancreas from SCN rhythms (13,88). In addition, a high-caloric diet has been shown to disrupt behavioural and molecular circadian rhythms in mice (89). Furthermore, two important regulators of homeostasis and metabolism in *Drosophila*, FOXO and GSK3b/Shaggy, were shown to be necessary for robust circadian rhythms (90,91), which emphasises the connection between metabolism and circadian clocks across the animal kingdom.

Collectively, the results from humans and animal models highlight the considerable involvement of the circadian machinery in metabolic pathways. A two-way interplay between these two systems is clear and the mechanisms governing their intercommunication are slowly emerging (Fig. 3).

## Disorders of Sleep Timing

The human population displays a wide spread of circadian phenotypes or chronotypes, with early types (larks) at one end of the spectrum and late types (owls) at the other. Chronotype is influenced by an individual’s genetics, development and exposure to light and dawn and dusk. In terms of the genetics, clock gene mutations can explain some of the differences in chronotype. Two recent large scale genomic studies identified variants in several clock-related loci (92,93), particularly *PER2*/3, underlying morningness in the general population. Different chronotypes can usually alter sleep patterns to accommodate both their social demands and circadian clock; Winston Churchill believed in the importance of good sleep, but was a very late chronotype and compensated with long afternoon naps (94). However, extreme misalignment with the external light-dark cycle leads to severely disrupted sleep-wake cycles, chronic fatigue and exhaustion. The underlying cause could be either deficits in core clock machinery leading to non-24h rhythms or deficits in the input pathways and entrainment systems that result in a misaligned rhythm. Examples of the first include delayed or advanced sleep phase disorders; Familial Advanced Sleep Phase syndrome is linked to mutations in *Per2* (95) and Familial Delayed Sleep Phase Syndrome to mutations in Casein Kinase 1 Delta (96) (Fig. 1). Recently, mutations in *Cry1* have been linked to Familial Delayed Sleep Phase syndrome, with a remarkably high frequency of 0.6% in the population, thereby affecting sleep in large numbers of individuals (97). In these conditions, due to a faster or slower molecular clock, the time window defined by the clock as optimal to sleep is shifted with respect to the external light-dark cycle, resulting in severe misalignment. In addition, situations where input pathways are deficient are also relatively common. Low levels of light within the nursing home environment result in circadian rhythm disruption (98) and patients with severe eye damage due to either genetic causes or trauma lose light input to the circadian clock resulting in severe misalignment (99). In these situations, behavioural rhythms imposed by care or feeding may help mask this disruption, but desynchronised and drifting peripheral clocks demonstrate the lack of entrainment which is manifest as poor and disrupted sleep.

## Treatment of Sleep and Circadian Rhythm Disruption (SCRD)

Despite our growing knowledge of the molecular mechanisms underlying the 24h circadian clock and its role in the development of chronic and debilitating diseases, there are limited therapeutic options available for the treatment of SCRD. As light is the primary zeitgeber for the SCN clock, bright light therapies and cognitive behavioural therapies that strengthen natural zeitgebers such as scheduled outdoor exercise (100,101) have been shown to have some success. However, potent pharmacological interventions are still lacking. Melatonin has long been characterised as an output of the circadian clock and can be used to modify the phase of the clock, presumably acting via the melatonin receptors that are expressed in the neurones of the SCN and multiple other cell populations across the body. Melatonin has therefore been studied as a possible chronotherapeutic drug and shows promise in certain circadian-related conditions (102,103). Prolonged release melatonin (tradename *Circadin*) is used to treat primary insomnia (104) in the aged and the agonist *Agomelatine* in the treatment of major depressive disorder (105). Most recently, *Tasimelteon* was approved in the United States in an orphan circadian disorder, non-24h sleep-sake disorder in the totally blind (106). Targeting the melatonin system, however, has limited efficacy; for example, Tasimelteon showed a beneficial effect on stabilising sleep-wake in 20% of the patient population after one month of treatment (106). As a consequence, recent efforts have focussed on developing alternatives, mainly targeting the core clock. Solt *et al.* reported a novel REV-ERBa receptor agonist was effective at regulating both sleep as well as metabolism in mice (6,107) and Hirota *et al.* have developed a small molecule Cryptochrome activator (108). An alternative strategy that has yet to be employed is the development of molecules that act on the light input pathway to the clock, providing a pharmacological replacement for light for the treatment of SCRD.

## Acknowledgements

The authors are very grateful for valuable input and critical comments from Prof. Andrea Nemeth and Dr. Jing Yu.

*Conflict of Interest statement.* None declared. 
